# Analysis of the Complete Genomes of Enterovirus 71 Subtypes in China

**DOI:** 10.1155/2021/5564099

**Published:** 2021-08-27

**Authors:** Lei Wang, Yuzhu Dai, Jun Cheng, Changgui Sun, Yu Chen, Dawei Cui

**Affiliations:** ^1^Department of Laboratory Medicine, The First Affiliated Hospital, Zhejiang University School of Medicine, Hangzhou 310003, China; ^2^Department of Laboratory Medicine, The 903 RD Hospital of PLA, 14 Lingyin Road, Hangzhou 310017, China; ^3^Key Laboratory of Clinical In Vitro Diagnostic Techniques of Zhejiang Province, Hangzhou 310003, China; ^4^Institute of Laboratory Medicine, Zhejiang University, Hangzhou 310003, China; ^5^Department of Blood Transfusion, The First Affiliated Hospital, Zhejiang University School of Medicine, Hangzhou 310003, China

## Abstract

Enterovirus 71 (EV-A71) is one of the most pathogens to hand, foot, and mouth disease (HFMD) as well as neurological complications in young children. Molecular characteristic of EV-A71 is important to prevent the virus outbreak. Here, the complete genomes of EV-A71 from China between 1998 and 2019 were downloaded from GenBank. The phylogenetic trees were developed by MEGA7.0 software, and the complete genetic epidemiological characteristics and amino acid mutations of EV-A71 from China were also analysed. The results showed that major epidemic EV-A71 subtype was C4b before 2004, while it turned to C4a after 2004 in mainland China, and C4 and B5 were major subtypes in Taiwan. VP1, VP4, 2C, 3C, 3D, and complete genome sequence can be used for virus genotyping, and VP1, VP4, and complete genomes have obvious advantages over other segments. There were many significant mutations in the viral complete genome sequence. This study indicated that the major C4 and B5 subtypes will contribute to the development of vaccines and drugs of EV-A71 for prevention and monitoring of EV-A71-associated HFMD in China.

## 1. Introduction

Hand, foot, and mouth disease (HFMD) is a common infectious disease that manifests as mouth pain, anorexia, low-grade fever, and small sores or ulcers on the hand, foot, and mouth. Children under the age of five are susceptible to HFMD [1]. Most patients recover spontaneously within a week, but a few cases can cause serious complications, such as myocarditis, pulmonary oedema, and aseptic meningoencephalitis [2]. More than 20 types of enterovirus can cause HFMD; coxsackievirus A16 (CoxA16) and enterovirus 71 (EV-A71) are the most common types among them [3].

EV-A71 was first isolated in 1969 and was identified as a new virus in 1974 [4]. Since then, EV-A71 has been widely prevalent worldwide, such as Asia, Europe, and America, and the Asia-Pacific region is the place where EV-A71 is the most widespread [5, 6]. EV-A71 was isolated from the Asia-Pacific region as early as the late 1990s [7]. After that, EV-A71 outbreaks were continuously reported in China, Malaysia, Cambodia, and Vietnam [8]. From 2008 to 2015, there were more than 13 million HFMD patients in China and 577,087 laboratory-confirmed cases (57,248 severe cases including 2,308 deaths), of which 73.8% of the confirmed severe cases and 92.5% of the deaths were caused by EV71 infection [9, 10]. Compared with other viruses, HFMD caused by EV-A71, in addition to routine clinical symptoms, is often accompanied by nervous system damage and even can lead to death [11]. It was reported that 90% of fatal cases of HFMD were due to EV-A71 infection [12]. EV-A71 has replaced poliovirus as the main viral pathogen of the central nervous system.

EV-A71 is a member of Picornaviridae, enterovirus genus, and belongs to human enterovirus A subtype [13]. The enterovirus genome is a single-stranded, positive-sense RNA of approximately 7.5 kb with a 22-amino-acid (aa) virus-encoded protein (VPg) covalently linked to the 5′ end and polyadenylated at its 3′ end. Flanked by 5′ and 3′ nontranslated regions (NTRs), the long open reading frame (ORF) encodes a large polyprotein that is processed into three primary precursors: one structural region (P1) and two nonstructural regions (P2 and P3) [14, 15]. P1 can be hydrolysed to 4 proteins (VP1, VP2, VP3, and VP4), P2 can be hydrolysed to 3 proteins (2A, 2B, and 2C), and P3 can be hydrolysed to 4 proteins (3A, 3B, 3C, and 3D) [16, 17].

At present, the pathogenesis of EV-A71 is not completely clear, although various study results for clinical patients indicate the general pathological process of virus infection [18]. Whether the difference in virulence of EV-A71 is due to some genetic characteristics of the virus itself, finally resulting in different clinical manifestations, still needs to be further discussed. Clinical treatment of EV-A71 infection is mainly supportive; although several drugs have been proven to be effective in the treatment of EV-A71, there is still a lack of a specific drug to treat EV-A71 infection [19]. This lack of treatment options also means that EV-A71 vaccination is the top priority. However, China still has not listed the EV-A71 vaccine as a national first-class vaccine at present, and the lack of public understanding of the EV-A71 vaccine has hindered the vaccination against EV-A71 to a certain extent [20]. Therefore, it is necessary to explore the molecular epidemiology of EV-A71 and analyse its virulence determinants, which could provide a theoretical basis for the research and development of related drugs and the prevention and treatment of EV-A71 infection. At present, there are many studies on EV-A71 evolutionary analysis, but most of them choose one segment of structural/nonstructural protein/gene sequence, but few studies have chosen several fragments, and less studies have analysed the complete genome sequences.

## 2. Materials and Methods

### 2.1. Study Design

#### 2.1.1. Sequence Collection and Preliminary Treatment

**Figure 1:**
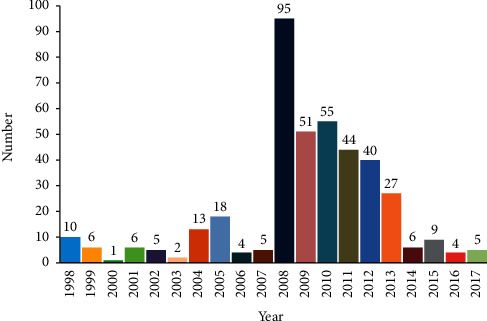
The number of EV-A71 isolated strains with the complete genome in different years in China. The height of the histogram represents the number of isolates, and the number on the histogram indicates the number of EV-A71 isolated strains.

**Figure 2:**
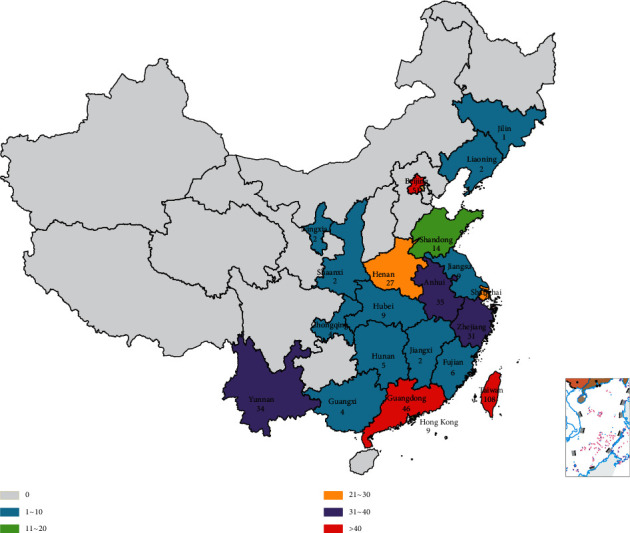
The number of EV-A71 isolated strains with the complete genome in different provinces of China. The EV-A71 isolated strains with a complete genome cover a total of 22 provinces. Orange represents less than 5 isolates, yellow represents between 5 and 12 isolates, lavender represents between 12 and 30 isolates, and dark purple represents greater than 30 isolates.

To assess the geographical distribution and genetic characteristics of the genes in the complete genomes of EV-A71 associated with HFMD in China, we performed an extensive genetic analysis using all available complete genomes of EV-A71 strains from China in the public database with the GenBank database of the National Center for Biotechnology Information (NCBI) website (http://www.ncbi.nlm.nih.gov/genbank). All the data of this study were downloaded from NCBI, which contains 451 strains (mainland China: 309 strains; Taiwan: 108 strains; Hong Kong: 9 strains) including 25 reference strains of 11 subtypes (A, B1–B5, and C1–C5) (Table 1) and 3 vaccine strains, covering 22 provinces in China over the period of 1998∼2017, although the data was collected up to Dec. 31, 2019, and the strains with complete genomes of EV-A71 were only collected from 1998 to 2017 (Figures 1 and 2).

### 2.2. Homology Analysis

BioEdit software (ver. 7.0.9.0; http://www.mbio.ncsu.edu/BioEdit/bioedit.html) was used for nucleic acid homology analysis. If the homology with EV-A71 from the same date and region/country of collection appeared to be 100%, only one strain was retained. Amino acid sequences of EV-A71 were compared for amino acid mutations by MEGA (ver. 7.0.26) software.

### 2.3. Phylogenetic Analysis

The software used in this study was MEGA (ver. 7.0.26) software. All sequences were ranked by ClustalW, constructing a phylogenetic tree with the neighbour-joining method; then bootstrap analysis was performed 1000 times.

### 2.4. Statistical Analysis

The proportion of different subtypes of EV-A71 strains in different provinces was compared by Chi-square test of SPSS (ver. 23.0) software.

## 3. Results

### 3.1. Phylogenetic Analysis Based on VP1 Gene Sequences of EV-A71

**Figure 3:**
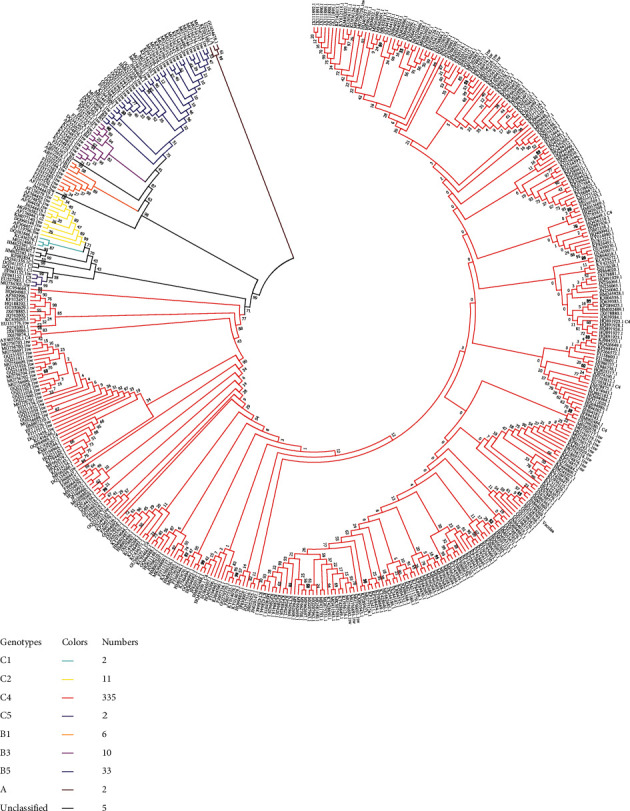
Phylogenetic tree constructed with the VP1 nucleotide sequences (891 bp) of EV-A71 strains with the complete genome. Red branches indicate genotype C4 strains; blue branches indicate genotype B5 strains; orange branches indicate genotype B1 strains; light blue branches indicate genotype C1 strains; yellow branches indicate genotype C2 strains; pink branches indicate genotype B4 strains; brown branches indicate genotype A strains; purple branches indicate genotype C5 strains. Each strain was named according to the GenBank accession number. Taiwan's strains have a TW suffix, reference strains have corresponding subtype name suffixes, and vaccine strains have the vaccine suffix.

According to the phylogenetic tree of VP1 (Figure 3), the strains isolated from mainland China were mainly the C4b subtype before 2004 and C4a after 2004. The EV-A71 strains with the C4a subtype in mainland China can be divided into two large groups and several small groups. No significant regional or temporal differences were found among these groups. The virus strains in Taiwan were more scattered on the phylogenetic tree and dominated by C4 and B5 subtypes. The C4 subtypes were distributed from 2004 to 2011, while the B5 subtypes appeared after 2008.

Except for Taiwan, C4 subtype was the major subtype of the EV-A71 strains in other provinces of China. We compare the differences among these provinces according to the proportion of C4 subtype strains. It was found that, among the 22 provinces, the strains are all C4 subtype in 15 provinces. We selected Anhui Province (including the most strains) as the representative to compare with the remaining 7 provinces and found that there were significant differences among the 5 provinces with Anhui Province (*p* < 0.05), which are Fujian, Hubei, Taiwan, Hong Kong, and Chongqing. Except for Taiwan, the number of strains in the other 4 provinces is less than 10, which makes 1 or 2 strains of other subtypes have a great influence on the proportion of subtype C4 strains. Therefore, we think that the differences among these provinces of the Chinese mainland are not significant. Although there are significant differences between the Chinese mainland and Hong Kong, there are fewer strains in Hong Kong. We still maintain the skepticism about the results. The difference between the Chinese mainland and Taiwan is obvious (*p* < 0.001). The results indicate that there are no obvious regional differences in the EV-A71 epidemic strains in mainland China, but there are notably differences between mainland China and Taiwan. The epidemic subtypes in mainland China have changed since 2004, which may be related to the transmission route of EV-A71 and the population mobility.

### 3.2. Phylogenetic Analysis Based on Other Gene Sequences of EV-A71

Surveillance of EV-A71 epidemic subtypes is one of the important ways to prevent EV-A71 outbreaks. Compared with the phylogenetic tree of each fragment, we could separate each subtype accurately by using the nucleic acid evolutionary tree of 3D, 3C, 2C, VP4, and the complete genomes than the VP1 nucleic acid phylogenetic tree, which indicate that these fragments and the complete genomes may be used for genotyping EV-A71.

**Figure 4:**
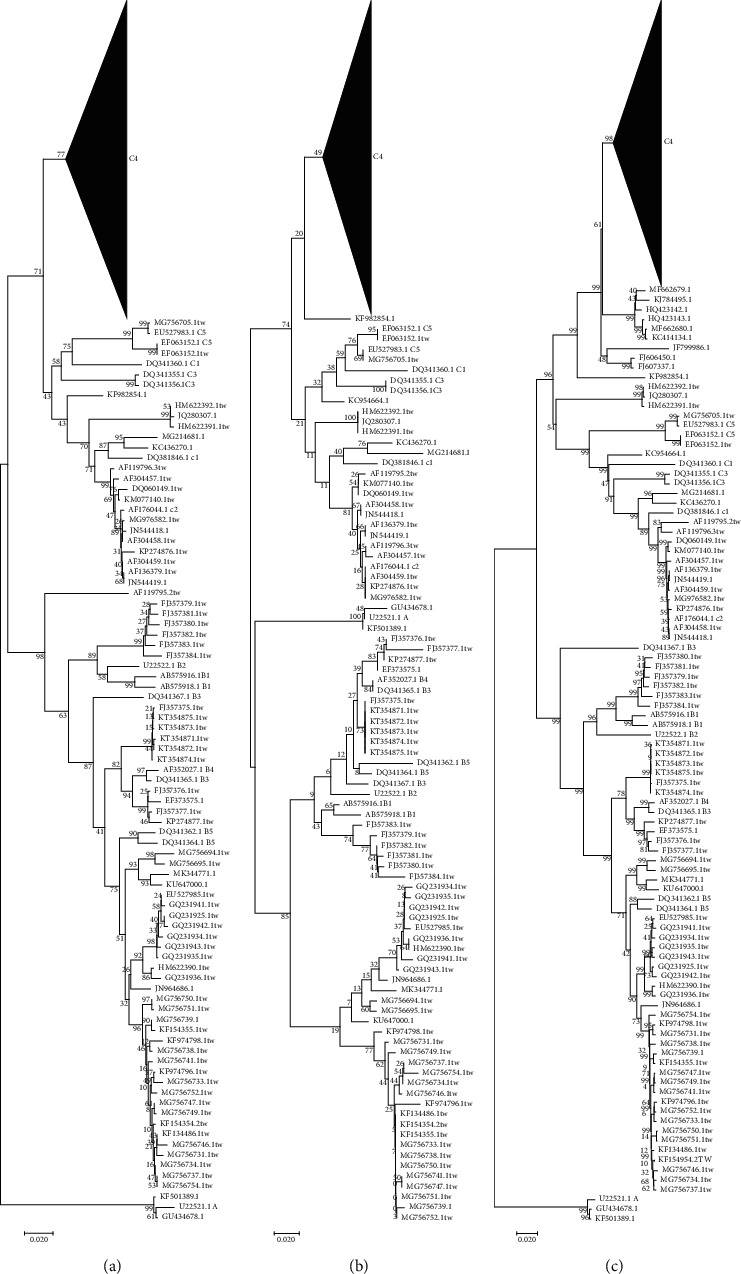
Phylogenetic trees constructed with the VP1, VP4, 2C, 3C, 3D, and whole-genome sequences of EV-A71. (a) VP1 phylogenetic tree; (b) VP4 phylogenetic tree; (c) whole genomes phylogenetic tree. The C4 subtree was compressed. Each strain is named according to the GenBank accession number. Taiwan's strains have a TW suffix, and the reference strains have corresponding subtype name suffixes.

**Figure 5:**
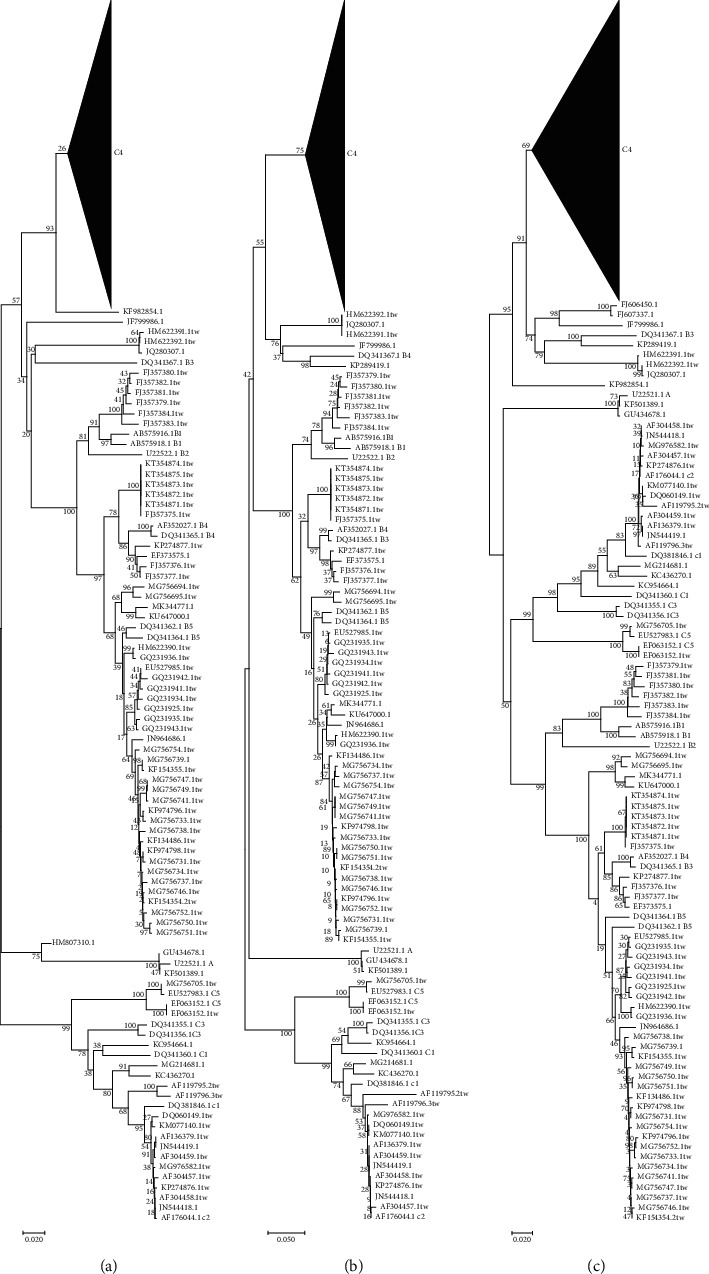
Phylogenetic trees constructed with 2C, 3C, and 3D of EV-A71. (a) 2C phylogenetic tree; (b) 3C phylogenetic tree; (c) 3D phylogenetic tree. The C4 subtree was compressed. Each strain is named according to the GenBank accession number. Taiwan's strains have a TW suffix, and the reference strains have corresponding subtype name suffixes.

In comparison with the phylogenetic tree of VP1, the phylogenetic trees based on VP4, 2C, 3C, 3D, and the complete genomes could distinguish each subtype, and there were some differences among them (Figures 4 and 5). In the VP1 phylogenetic tree, we found that 5 strains (KF982854.1, HM622391.1, HM622392.1, AF119795.2, and JQ280307.1) could not be directly typed in the phylogenetic tree, which accounts for 1.10% among the 451 strains. The KC954664.1 strain could not be classified with the VP4 phylogenetic tree, while it was classified into the C4 subtype with the VP1 phylogenetic tree, and the AF119795.2 strain was classified into the C2 subtype in the VP4 phylogenetic tree. GQ994992.1 could not be classified intuitively in the VP4 phylogenetic tree, which was classified as the C4 subtype in the VP1 phylogenetic tree, and other typing results were the same in the VP1 phylogenetic tree. In the 2C phylogenetic tree, AF119795.2 was divided into C1 subtype, and KC954664.1 was also divided into C1 subtype, which was classified into C4 subtype in the VP1 phylogenetic tree. KF982854.1 was classified into subtype C4, JQ280307.1 was classified into subtype B3, and HM807310.1 was classified into type A which was originally subtype C4. In the 3C phylogenetic tree, JF199986.1 and KP289429.1 were classified into the B3 subtype, and these strains were classified into the C4 subtype in the VP1 phylogenetic tree. In addition, JQ280307.1 was also classified into the B3 subtype, KC954664.1 was classified into the C3 subtype, and AF119795.2 was classified into the C2 subtype. In the 3D phylogenetic tree, the reference strain DQ341367.1 was not divided into the same branch of other reference strains of type B, and 5 strains were divided into the same subtype of the reference strain DQ341367.1. KC954664.1 was classified into subtype C1, and AF119795.2 was classified into subtype C2. In the phylogenetic tree of the complete genome, KC954664.1 was divided into the C1 subtype, and AF119795.2 was divided into the C2 subtype.

### 3.3. Sequence Homology Comparison of Each Gene Fragment

The homology of the different gene regions of VP4, VP2, VP3, VP1, 2A, 2B, 2C, 3A, 3B, 3C, 3D, and the coding region was 76.8%∼100%, 77.9%∼100%, 77.9%∼100%, 80.9%∼100%, 93.3%∼100%, 69.3%∼100%, 77%∼100%, 71.7%∼100%, 63.6%∼100%, 69.3%∼100%, 75%∼100%, and 78.7%∼99.9%, respectively (Table 2).

### 3.4. Amino Acid Mutation Analysis

Using the U22521.1 strain as the reference strain, 70 mutation sites were found in the 2A segment, 51 sites in the 2B segment, 143 sites in the 2C segment, 50 sites in the 3A segment, 11 sites in the 3B segment, 91 sites in the 3C segment, and 209 sites in the 3D segment. Additionally, 116 mutation sites were found in the VP1, 108 mutation sites in the VP2, 67 mutation sites in the VP3, and 34 mutations sites in the VP4 (Table 3). Comparing the occurrence frequency of mutations on the various sites of the complete genomes each year, we found that there were significant differences (*p* < 0.001) (Table 4). The frequency of mutations on the 57 sites of 2A reached 100% in 1999 and 2000, which gradually decreased. In 2003∼2004, it even disappeared. In 2005, it reappeared and remained above 70% since 2006. The difference in frequency between 1998∼2004 and 2005∼2017 is statistically significant (*p* < 0.001). The frequency of mutations at the 2A-68 was the highest among these years, but it was low in 2004 to 2006. There is a statistical difference in the mutation frequency between these three years and other years (*p* < 0.001). Although mutations at the 2C-41 site existed before 2006, the frequency of occurrence was low and even disappeared in some years, and it suddenly increased in 2007, which reached 60%. Although it decreased in 2008, the frequency remained above 80% after 2009. The frequency of mutations at the 3A-47 site was low from 1999 to 2006, but it increased rapidly from 2007 to 2010. The frequency of mutations at the 3B-15 had a peak in 2001–2002, disappeared in 2003, and then reappeared in 2004, which reached 92.3%. There is a statistical difference in the mutation frequency between 1998∼2003 and 2004–2017 (*p* < 0.001). The sites of 3C-49, 3D-33, 3D-263, and VP2-144 were similar to the 3B-15 site. The mutations of 3C-158 disappeared in 1999∼2000, reappeared in 2001, reached a frequency of 100% in 2004∼2005, fluctuated in the range of 44.4%∼96.4% in 2006∼2015, and disappeared again in 2016∼2017. There were some differences in 2001∼2015, 1998∼2000, and 2016∼2017 (*p* < 0.001), but there was no significant difference between 1998∼2000 and 2016∼2017 (*p*=0.274).

## 4. Discussion

Compared with the VP1 phylogenetic tree, the complete genome phylogenetic tree was closest to it, and there were 3, 5, 5, and 8 strains in the VP4, 3C, 2C, and 3D phylogenetic tree which were different, respectively. Interestingly, when we used the full-gene phylogenetic tree as a reference, there were 2, 2, 4, 4, and 6 strains in the VP4, VP1, 2C, 3C, and 3D phylogenetic tree, which were different from it. The homology of the subtypes with B5 and C4, which contained most virus strains in the typing results of different fragments in this study, was as follows. When typing with VP1 fragments, we found that the homology of VP1 fragments within the C4 subtype was more than 92.1%, while it was less than 90.3% among the C4 subtype and other reference sequences. The lowest homology between B5 subtype strains and B5 subtype reference strains was 94.5%. The highest homology within the reference strains of other subtypes was 92.3%, which is not completely consistent with our conclusion that those with VP1 homology greater than 92% are the same subtype and that those less than 92% are different subtypes [13]. The homology between the strains of the B5 subtype and the reference strains of the B3 and B4 subtypes was also high, both of which were more than 90%. When typing with the complete genomes, we found that the full gene homology between the C4 subtype and the reference strains of the same subtype was 89.5% to 99.9%, while the homology between the C4 subtype and other reference sequences was 78.6% to 84.7%. The homology among the B5 subtype and the B5 subtype reference strains was 93.6% to 96.8%, and the homology among the B5 subtype and other subtype reference strains was 79.3% to 92.8%. We believe that when assessing the homology of the complete genomes, the dividing line should be at 93%.

When typing with VP4 fragments, the homology was between 88.8% and 100% among the C4 subtype, while the homology between the C4 subtype and other subtypes was 76.8%∼89.8%. There was some overlap between the two ranges. When analysing the homology of the VP4 fragments from the B5 subtype, there was a great overlap between the B5 subtype and the reference strains of each subtype, so it was impossible to distinguish the subtype by homology. We think that it may be due to the relatively short nature of the VP4 fragment, which is only 207 bp; therefore, the mutation has a relatively greater impact on it than the longer fragments. When typing with 2C fragments, the homology within the C4 subtype was between 83.9% and 100%, and the homology between the C4 subtype and other subtype reference strains was between 76.5% and 85.4%. The intrasubtype homology of the B5 subtype was between 94.5% and 98.1%. Although there was no overlap between the intrasubtype and intersubtype homology of the B5 subtype, there was an overlap when comparing with the homology of the C4 subtype.

When typing with the 3C fragments, the homology within the C4 subtype was 89.6%∼100%, and the homology among the C4 subtype and other subtype reference strains was 73.4%∼83.9%. The homology within the B5 subtype was 93.9%∼97.4%, and the homology among the subtypes was 73.5%∼92.1%. The situation is similar to the 2C segment. When 3D fragments were used for typing, the homology within the C4 subtype was 84.5%∼100%, and the homology among the C4 subtype and other subtype reference strains was 75.6% to 84.8%. Four strains within the C4 subtype were low homology with C4 subtype reference strains, ranging from 84.5% to 86.9%. Comparing the above results, we found that using the complete genomes phylogenetic tree to type EV-A71 viruses is the best choice. The gap between the complete genomes and other fragment phylogenetic trees was the smallest, and the difference between the intrasubtype homology and intersubtype homology was obvious. In addition, the complete genomes contain all the mutation information, so the phylogenetic tree with the complete genomes can significantly reflect the mutation of the virus genome. Although the evolutionary tree of complete genomes for EV-A71 has the above advantages, the whole gene sequence is too long, which requires different fragments to be sequenced and spliced. The workload of sequencing and splicing will be much larger. Although the typing result of the evolutionary tree of complete genomes is the best, it is not suitable for the greatest priority typing scheme under certain conditions. However, the gaps among the VP4, VP1, and the phylogenetic tree of complete genomes are small, and the gene length is much shorter for the fragments. VP4 has the advantages of short fragments and low sequencing effort, while VP1 is the only fragment with no overlap of homology within and between subtypes (without using reference strain AY465356.1). A clear dividing line can be established, and subtypes can be distinguished by homology differences. Although the gap between the 3C fragment phylogenetic tree and the phylogenetic tree of complete genomes is relatively large, the homology gap within and between subtypes is also obvious, and it is also a favourable fragment for typing. There are 4 different strains between the 2C fragment phylogenetic tree and phylogenetic tree of complete genomes, but the homology range of intrasubtype and intersubtype is greater than the 3C fragment, and the length of the 2C fragment is longer than the 3C fragment, so typing with the 3C fragment should be more favourable than the 2C fragment. The difference between the phylogenetic tree of 3D fragment and the complete genome was the largest, and the homology difference within and between subtypes was the least. The length of the 3D fragment was the longest among all fragments, so we think that although the 3D fragment can be used for genotyping, priority should be given to typing with other fragments or whole genomes.

Using the U22521.1 strain as a reference, we analysed the amino acid variation in Chinese EV-A71 strains. Chang et al. found that neutralizing monoclonal antibodies could specifically bind amino acid residues containing VP1-240 to VP1-260 [21]. Most of the Chinese strains, including vaccine strains, have S240T, E244K, S246P, and I249V mutations; the mutations including VP1-S240I, VP1-Y245K, VP1-M255I, VP1-K256E, and VP1-V258I are present in 1 strain; 4 strains have the VP1-E244N mutation; the prevention effect of vaccines against these nine EV-A71 strains may be weaker than that against other strains. In another study, Lin et al. found three well-identified EV-A71 linear neutralizing epitopes in capsid proteins: VP1 amino acids 163∼177 and 208∼222, and VP2 amino acids 136∼150 [22]. We found 28 mutations in the VP1 163∼177 and 208∼222 regions, but the number is much larger in the VP2 136∼150 region, especially at the VP2-144 site. These results indicate that the fragment of VP1 may be more suitable for the development of novel vaccines. Jiang et al. showed that VP3 EV-A71-176∼190 can be used for effective recombinant gene vaccine research and development, and this study found that 10 strains carried mutations in this segment [23]; whether these mutations will affect vaccine research and development has not been confirmed presently.

Regarding the receptor related to EV-A71 infection, we also found that the 273 strains had VP1-G/Q145E mutations, with 83.9% in mainland Chinese mutant strains and 72.2% in Taiwan mutant strains, and 4 strains carried VP1-K244N. Both of these mutations can lead to changes in protein spatial structure and cause the EV-A71 virus to lose the ability to bind to the PSGL-1 receptor, which is closely related to cell invasion [24, 25]. Regarding VP1-145, another study showed that VP2 (149M) and VP1 (145E) mutations cooperatively promote EV-A71 virus binding and RNA accumulation, contributing to viral infectivity in vitro and mouse lethality in vivo [26]. We found that only 2 strains had both mutations. Another mutation (VP1-A170 V) may lead to virulence enhancement [27]; we also found only 1 strain carrying the mutation. Whether these strains will show similar characteristics when infecting humans needs to be further proved. There is another receptor (SCARB2) playing an important role in virus uncoating, and Chen et al. identified that EV-A71 binds to SCARB2 via a VP1 cleft around residue Gln-172, especially residues Gln-152, Arg-166, Trp-171, Gln-172, Thr-173, Thr-175, Asn-176, Ser-178, Phe-180, and Arg-236 [28]. We found only 1 strain that had a mutation at site 166 and another strain that had a mutation at site 172. The result also proved the importance of SCARB2 in virus uncoating and infectivity.

Zhang et al. showed that there were no significant differences in neurovirulence between genotype B and genotype C strains. Interestingly, they found that the mutations E145 G/Q, E164D/K, and T292N/K of subtype B strains were closely related to neurological complications. In the case of genotype C, the N31D mutation increased the risk for nervous system complications, whereas the I262V mutation decreased the risk of nervous system complications [29]. There were 17 strains of genotype C with N31D mutation, and 12 strains of genotype C had the I262V mutation in our study. The strains containing N31D mutations were mostly isolated in 1998 and 2008, which may be related to the outbreaks of HFMD in Taiwan and Anhui in 1998 and 2008. Mutated residues (27S, 31S/D, 98K, 145G/Q, 164E, and 240A/S) might be potential virulence determinants in VP1 of EV-A71, and a total of 117 strains in China have these mutations [30], which could explain that EV-A71 infection usually causes neurological complications. We found that 300 strains have VP1-T289A mutations. At the same time, we compared the changes in the mutation detection by year and found that in 1998 to 2003 and in 2006 to 2017, the mutant strain ratio was more than 60% and up to 100%, respectively; from 2004 to 2005, it was relatively low, at less than 20%. According to Guan's report, viruses with T289 A mutations have an increased risk for nervous system complications [31].

Using synthetic linear peptide sequence filter epitopes in the VP1 region, Lim et al. found that the conserved neutralization epitope consisted of residues 215∼219 of VP1 [32]. Ku et al. found that neutralizing antibodies bound to amino acid residues 211∼224 in the GH loops of VP1 [33]. Liu et al. found that the neutralizing epitope of residues 136∼150 located in VP2 may contribute to the virus's ability to infect rodent cells, amino acid residues 211∼220 of VP1 could specifically react with monoclonal neutralizing antibodies, and residues of the VP2 region could react with antibodies against other subtypes [34]. In another study, the VP2 epitope (aa141-155) was immunodominant, and a broad-spectrum vaccine strategy targeting the high-affinity epitope of the VP2 EF loop may elicit effective immune responses against EV-A71 infection [35]. In this study, we found that EV-A71 strains in China have 2 mutations at residues 212∼219 of VP1, but there are many mutations in 136∼155 of VP2, suggesting that 212∼219 of VP1 is highly conserved and is a suitable site for recombinant vaccine development. Chen et al. found that most neutralizing anti-EV-A71 monoclonal antibodies are specific to conformational epitopes [36]. Furthermore, Kiener et al. found that the mutations P59L, A62D, A62P, and E67D could abolish both monoclonal antibody binding and neutralization activity [37]. We found that a total of 5 strains in China had mutations in the three sites. These results indicated that the monoclonal antibody against linear epitope had a good protective effect on Chinese people, and genetic engineering vaccine maybe had a good application prospect in China. A total of 10 human anti-EV-A71 IgM epitopes (40∼51 in VP1; 16∼27, 61∼72, 118∼129, and 148∼159 in VP2; and 28∼39, 34∼45, 43∼54, 70∼81, and 223∼234 in VP3) were identified in acute-phase sera [38]. We found that epitope residues 61∼72aa in VP2 had the least mutations among the 4 epitopes of VP2 and that residues 43-54aa in VP3 had the least mutations among the 5 epitopes of VP3. However, the serine to threonine mutation at VP2-144 present in recently emerged EV-A71-C4 Chinese strains abolished antigenicity, and that mice injected with this virus strain did not produce any antibodies against the VP2 protein [39]. We found that most strains had the VP2-S144T mutation after 2008, and in 2016 and 2017, the prevalence of this mutation was up to 100%. In another study, Huang et al. found that VP2-N143D, VP1-H116Y, D167E, K18R, and S275A were possibly associated with antigenic variation [40]. Peptides widely distributed in VP2 are related to cellular immunity [41]. We found most strains mutated in these sites. In conclusion, using the epitope of residue 43∼54 in VP3 to detect EV-A71 infection may be more effective than other epitopes. There are two drugs acting on the early stage of EV-A71 infection, but all strains in China have a VP3-R227K mutation in the capsid protein, which was determined to be a mutation conferring resistance to AN-12-H5 and AN-23-F6 [42]. This finding suggests that AN-12-H5 and AN-23-F6 may not be suitable for the Chinese.

We analysed amino acid mutations in nonstructural proteins using U22521.1 as a reference. As reported, the His21-Asp39-Cys110 triplet in 2A is the key active enzyme site for cutting eukaryotic translation initiation factor 4G [43]. In this study, we found no mutation in the three sites, possibly because the 2A protein (2A^pro^) plays a critical role in the process of the virus's genetic modification, transcription, translation, and protein modification [44,45]. Mutations in these 3 sites may disable the replication of the virus. After mutation in residues 146∼149 of the 2A protein, the virus loses transcriptional activator activity but does not lose the enzymatic activity of 2A protein [46]. In this study, 3 strains were mutated in residues 146–149 of 2A. We found that Chinese 266 strains encoded a Val at P814, 392 strains encoded Val at P1148, and 407 strains encoded Ala at P1728. These strains had an enhanced probability of neurovirulence, which was reported [47].

The 3A protein is a small hydrophobic protein that contains a C-terminal hydrophobic anchor that is responsible for its membrane association [48]. Itraconazole (ITZ) is an effective inhibitor at low concentrations of EV-A71 replication, and Gao et al. confirmed that resistance to ITZ was carried in nonstructural protein 3A (3A V51L and 3A V75A) [49]. We found that 1 strain had the same mutation, but 7 strains had V51I, 1 strain with V75M mutations, and 1 strain had V75G mutations; however, whether these mutations conferred resistance to ITZ was not confirmed.

3C^pro^ is EV-A71's key molecule combating the host's innate immune response [50], and the 3C–H40D mutation can obviously decrease the activity of the protease [51]. We found no mutations at this site. It proves the importance of 3C protein to the virus, and we can develop drugs for the treatment of EV-A71 infection based on 3C protein. Chen et al. showed that 3C residues 45–52 involved in the ubiquitin-conjugating enzyme 9 (Ubc9) interaction contributed to 3C sumoylation potential and protein stability regulation [52]. We found a total of 297 strains carrying mutations located at 3C-49, and 9 strains had 3C–V46A mutations. The mutation rate of C4a in 3C-49 was 80.2%, while the mutation rate of C4b in this site was 10%. It seems that the mutation located at 3C-49 is related to the genotype of strains.

Regarding the 3D polymerase, an RNA-dependent RNA polymerase (RdRp) of EV-A71, the 3D-T251I/V mutation can reduce the ability of EV-A71 to replicate in vitro and virulence at 39.5°C [53]. We found 62 strains with 3D-251 mutated to Ile or Val which contains 13 B5 subtype strains. These 13 viruses may be used in the development of live attenuated vaccines. Studies found a novel site (site 311 located at the base of the palm domain of EV-A71 3D polymerase (3D^pol^)) that is essential for EV-A71 VP uridylylation as well as viral replication [54]. It is a potential site for antiviral development. We found 3 strains carrying mutations located at this site, which were all isoleucine. Interestingly, this mutation should be fatal to the virus, but perhaps because of the similarity between leucine and isoleucine, these strains survived. Comparing the 3 strains and other isolates, we found that these 3 virus strains had the F337Y mutation, while in the other strains, only 3 isolates had the same mutation. Whether there is a connection between these 2 mutations requires additional experiments to prove. Liu et al. demonstrated that the 3D protein is modified by small ubiquitin-like modifier 1 (SUMO-1) both during infection and in vitro. Residues K159 and L150/D151/L152 were responsible for 3D sumoylation [55]. We found no mutations in these sites. This proved that these four residues were very important for the activity of the 3D protein, and drugs for the treatment of EV-A71 infection could be developed based on these four residues. As a drug acting on 3D^pol^, DTriP-22(4{4-[(2-bromo-phenyl)-(3-methyl-thiophen-2-yl)-methyl]-piperazin-1-yl}-1-pheny-1H-pyrazolo [3,4-d]pyrimidine) can inhibit the poly (U) elongation activity by preventing nucleotide access to the cavity of 3D^pol^ [56]. The 3D polymerase drug resistant site is Arg163 [57]. We found that the residues of all isolates in this site are Arg; DTriP-22 should have favourable application prospects in China. The 3D polymerase has palm, thumb, and finger protein domains [58].

### 4.1. Limitations

This study is a retrospective study; it has some limitations. Firstly, the whole gene sequences of EV-A71 in China analysed in this study were downloaded from the public database with the GenBank database of the National Center for Biotechnology Information (NCBI) website (http://www.ncbi.nlm.nih.gov/genbank), but not from our own laboratory. Secondly, there were only 436 EV-A71 strains in China, which did not cover all the provinces in China, meaning that they possibly missed some strains isolated from other genotypes. Thirdly, although we found a sudden increase or decrease in mutations at several sites in 2007 or 2008, we did not verify whether the mutations at these sites were related to the outbreaks of HFMD in 2007 and 2008. Nevertheless, this study is based on the whole gene sequence at the molecular level, which helps us understand the genetic characteristics and evolution of HFMD-related EV-A71 strains circulating in China from 1998 to 2017. To further clarify the detailed genetic characteristics and evolution of EV-A71 strains, the research scope should be expanded, and the sample size should be increased in the future to provide a scientific basis for the prevention and treatment of hand, foot, and mouth disease in China.

## 5. Conclusions

Generally, the evolution rate of EV-A71 strains in China is not fast, and the isolated subtypes are relatively concentrated, C4a subtype is the most subtype of EV-A71 in mainland China, and C4 and B5 subtypes are the major subtypes in Taiwan of China. VP1, VP4, 2C, 3C, 3D, and complete genomes can be used for virus genotyping, VP1, VP4, and whole genomes are more favourable than other fragments. These findings provide useful information for further understanding the evolutionary relationship and genetic characteristics of EV-A71 strains in China and for vaccine development and virulence variation research in the future. Therefore, it is necessary to carry out long-term and continuous monitoring of EV-A71 to provide a basis for the study of an improved vaccine and the control and prevention of HFMD in China.

## Figures and Tables

**Table 1:** Reference strains of EV-A71 subtypes in this study.

Subtypes	Reference strains		
A	U22521.1		
B1	AB575916.1	AB575918.1	
B2	U22522.1		
B3	DQ341365.1	DQ341367.1	
B4	AF352027.1		
B5	DQ341362.1	DQ341364.1	
C1	DQ381846.1	DQ341360.1	
C2	AF176044.1		
C3	DQ341355.1	DQ341356.1	
C4	EU703814.1	FJ606449.1	GQ994989.1
	GU366191.1	GU396280.1	HQ456309.1
	HQ891925.1	AY465356.1	
C5	EF063152.1	EU527983.1	

**Table 2:** Proportion of C4 subtypes of EV-A71 strains in each province.

Province	Number of C4 subtype strains	Number of virus strains	Proportion of C4 subtype strains
Anhui	35	35	100%
Beijing	49	51	96.07%
Fujian	4	6	66.67%
Guangdong	27	27	100%
Guangxi	4	4	100%
Henan	27	27	100%
Hubei	7	9	77.78%
Hunan	5	5	100%
Jilin	1	1	100%
Jiangsu	9	9	100%
Jiangxi	2	2	100%
Liaoning	2	2	100%
Ningxia	2	2	100%
Shandong	14	14	100%
Shaanxi	2	2	100%
Shanghai	26	26	100%
Shenzhen	19	19	100%
Taiwan	47	108	43.52%
Hong Kong	8	9	88.89%
Yunnan	33	34	97.06%
Zhejiang	31	31	100%
Chongqing	3	4	75%

**Table 3:** Comparison of the nucleotide and amino acid identities of the Chinese EV-A71 strains.

	Sequence homology range (%)	Amino acid mutation site	Length of amino acid sequence	Rate of mutation (%)
VP4	76.8–100	34	69	49.3
VP2	77.9–100	108	254	42.5
VP3	77.9–100	67	242	27.7
VP1	80.9–100	116	297	39.1
2A	93.3–100	70	150	46.9
2B	69.3–100	51	99	51.5
2C	0.77–100	143	329	43.5
3A	71.7–100	50	86	58.1
3B	63.6–100	11	22	50
3C	69.3–100	91	183	49.7
3D	0.75–100	209	462	45.2
Coding regions	78.7–99.9	950	2193	43.3

**Table 4:** Comparison of the amino acid substitutions in the Chinese EV-A71 strains in different years.

Amino acids mutation ratio										
Year	2A-57	2A-68	2C-41	3A-47	3B-15	3C-49	3C-158	3D-33	3D-263	VP2-144
1998	0.000	0.800	0.100	0.800	0.200	0.000	1.000	0.000	0.200	0.000
1999	1.000	1.000	0.000	0.000	0.000	0.000	0.000	0.000	0.000	0.000
2000	1.000	1.000	0.000	0.000	0.000	0.000	0.000	0.000	0.000	0.000
2001	0.500	0.500	0.333	0.333	0.500	0.167	0.500	0.500	0.333	0.333
2002	0.200	0.400	0.000	0.000	0.600	0.200	0.800	0.000	0.400	0.000
2003	0.000	0.500	0.000	0.000	0.000	0.000	0.500	0.000	0.000	0.000
2004	0.000	0.077	0.000	0.077	0.923	0.846	1.000	0.000	0.077	0.000
2005	0.056	0.111	0.056	0.111	0.778	0.889	1.000	0.000	0.000	0.000
2006	0.500	0.000	0.000	0.000	0.750	1.000	0.750	0.750	0.000	0.000
2007	0.800	0.600	0.600	0.200	0.600	0.800	0.800	0.600	0.200	0.200
2008	0.768	0.600	0.474	0.400	0.789	0.695	0.800	0.589	0.558	0.495
2009	0.725	0.706	0.686	0.549	0.745	0.608	0.902	0.725	0.412	0.706
2010	0.945	0.873	0.873	0.873	0.982	0.855	0.964	0.945	0.873	0.945
2011	0.932	0.932	0.864	0.818	0.841	0.795	0.932	0.841	0.727	0.841
2012	0.900	0.975	0.950	0.650	0.600	0.500	0.575	0.550	0.550	0.575
2013	0.926	1.000	0.889	0.963	0.667	0.667	0.556	0.889	0.704	0.926
2014	0.889	0.972	0.917	0.889	0.889	0.806	0.639	0.889	0.722	0.917
2015	0.889	1.000	1.000	0.778	0.667	0.667	0.444	0.556	0.667	0.778
2016	1.000	1.000	1.000	0.750	1.000	0.750	0.000	1.000	1.000	1.000
2017	1.000	1.000	1.000	1.000	1.000	1.000	0.000	0.800	1.000	1.000

## Data Availability

The data used to support the findings of this study are included within the article.

## References

[1] Xu J., Yang M., Zhao Z (2020). Meteorological factors and the transmissibility of hand, foot, and mouth disease in Xiamen city, China. *Frontiers of Medicine*.

[2] Lulla V., Dinan A. M., Hosmillo M. (2019). An upstream protein-coding region in enteroviruses modulates virus infection in gut epithelial cells. *Nature Microbiology*.

[3] Yang B., Lau E. H. Y., Wu P., Cowling B. J. (2016). Transmission of hand, foot and mouth disease and its potential driving factors in Hong Kong. *Scientific Reports*.

[4] Van Tu P., Thao N. T. T., Perera D. (2007). Epidemiologic and virologic investigation of hand, foot, and mouth disease, southern Vietnam, 2005. *Emerging Infectious Diseases*.

[5] Chang H.-L., Chio C.-P., Su H.-J. (2012). The association between enterovirus 71 infections and meteorological parameters in Taiwan. *PLoS One*.

[6] NikNadia N., Sam I.-C., Rampal S. (2016). Cyclical patterns of hand, foot and mouth disease caused by enterovirus A71 in Malaysia. *PLoS Neglected Tropical Diseases*.

[7] Ho M., Chen E.-R., Hsu K.-H. (1999). An epidemic of enterovirus 71 infection in Taiwan. *New England Journal of Medicine*.

[8] Komatsu H., Shimizu Y., Takeuchi Y., Ishiko H., Takada H. (1999). Outbreak of severe neurologic involvement associated with enterovirus 71 infection. *Pediatric Neurology*.

[9] Zhu Z., Zhu S., Guo X (2010). Retrospective seroepidemiology indicated that human enterovirus 71 and coxsackievirus A16 circulated wildly in central and southern China before large-scale outbreaks from 2008. *Virology Journal*.

[10] Yang B., Liu F., Liao Q. (2017). Epidemiology of hand, foot and mouth disease in China, 2008 to 2015 prior to the introduction of EV-71 vaccine. *Euro Surveillance*.

[11] Chong C. Y., Chan K. P., Shah V. A (2003). Hand, foot and mouth disease in Singapore: a comparison of fatal and non-fatal cases. *Acta Paediatrica*.

[12] Xing W., Liao Q., Viboud C. (2014). Hand, foot, and mouth disease in China, 2008-12: an epidemiological study. *The Lancet Infectious Diseases*.

[13] Wang Y., Li Y., Yang Y. (2020). Virological investigation of genetic variation of enterovirus type 71 in hand, foot and mouth disease. *Experimental and Therapeutic Medicine*.

[14] Chong P., Liu C.-C., Chow Y.-H., Chou A.-H., Klein M. (2015). Review of enterovirus 71 vaccines. *Clinical Infectious Diseases*.

[15] Muslin C., Kain A., Bessaud M., Blondel B., Delpeyroux F. (2019). Recombination in enteroviruses, a multi-step modular evolutionary process. *Viruses*.

[16] Brown B. A., Pallansch M. A. (1996). Complete nucleotide sequence of enterovirus 71 is distinct from poliovirus. *Virus Research*.

[17] Wang L. C., Chen S. O., Chang S. P., Lee Y. P., Lin C. F. (2015). Enterovirus 71 proteins 2A and 3D antagonize the antiviral activity of IFN-*γ* via signaling attenuation. *Journal of Virology*.

[18] Klein M. H. (2015). EV71 vaccines: a first step towards multivalent hand, foot and mouth disease vaccines. *Expert Review of Vaccines*.

[19] Ren P., Zou G., Bailly B. (2014). The approved pediatric drug suramin identified as a clinical candidate for the treatment of EV71 infection—suramin inhibits EV71 infection in vitro and in vivo. *Emerging Microbes and Infections*.

[20] Fu H., Zhang Z., Dai Y., Liu S., Fu E. (2020). Brequinar inhibits enterovirus replication by targeting biosynthesis pathway of pyrimidines. *American Journal of Tourism Research*.

[21] Chang G.-h., Luo Y.-j., Wu X.-y., Si B.-y., Lin L., Zhu Q.-y. (2010). Monoclonal antibody induced with inactived EV71-Hn2 virus protects mice against lethal EV71-Hn2 virus infection. *Virology Journal*.

[22] Li Y.-X., Zhao H., Cao R.-Y. (2014). Recombinant tandem multi-linear neutralizing epitopes of human enterovirus 71 elicited protective immunity in mice. *Virology Journal*.

[23] Jiang L., Fan R., Sun S. (2015). A new EV71 VP3 epitope in norovirus P particle vector displays neutralizing activity and protection in vivo in mice. *Vaccine*.

[24] Lin H.-Y., Yang Y.-T., Yu S.-L. (2013). Caveolar endocytosis is required for human PSGL-1-mediated enterovirus 71 infection. *Journal of Virology*.

[25] Nishimura Y., Lee H, Hafenstein S (2013). Enterovirus 71 binding to PSGL-1 on leukocytes: VP1-145 acts as a molecular switch to control receptor interaction. *PLoS Pathogens*.

[26] Huang S.-W., Wang Y.-F., Yu C.-K., Su I.-J., Wang J.-R. (2012). Mutations in VP2 and VP1 capsid proteins increase infectivity and mouse lethality of enterovirus 71 by virus binding and RNA accumulation enhancement. *Virology*.

[27] Mcminn P., Stratov I., Nagarajan L., Davis S. (2001). Neurological manifestations of enterovirus 71 infection in children during an outbreak of hand, foot, and mouth disease in western Australia. *Clinical Infectious Diseases*.

[28] Chen P., Song Z., Qi Y. (2012). Molecular determinants of enterovirus 71 viral entry. *Journal of Biological Chemistry*.

[29] Zhang B., Wu X., Huang K. (2014). The variations of VP1 protein might be associated with nervous system symptoms caused by enterovirus 71 infection. *BMC Infectious Diseases*.

[30] Liu Y., Fu C., Wu S. (2014). A novel finding for enterovirus virulence from the capsid protein VP1 of EV71 circulating in mainland China. *Virus Genes*.

[31] Guan D., van der Sanden S., Zeng H (2012). Population dynamics and genetic diversity of C4 strains of human enterovirus 71 in Mainland China, 1998-2010. *PLoS One*.

[32] Lim X. F., Jia Q., Khong W. X (2012). Characterization of an isotype-dependent monoclonal antibody against linear neutralizing epitope effective for prophylaxis of enterovirus 71 infection. *PLoS One*.

[33] Ku Z., Ye X., Huang X (2013). Neutralizing antibodies induced by recombinant virus-like particles of enterovirus 71 genotype C4 inhibit infection at pre- and post-attachment steps. *PLoS One*.

[34] Liu C.-C., Chou A.-H., Lien S.-P. (2011). Identification and characterization of a cross-neutralization epitope of Enterovirus 71. *Vaccine*.

[35] Xu L., He D., Li Z. (2014). Protection against lethal enterovirus 71 Challenge in mice by a recombinant vaccine candidate containing a broadly cross-neutralizing epitope within the VP2 EF loop. *Theranostics*.

[36] Chen Y., Li C., He D. (2013). Antigenic analysis of divergent genotypes human Enterovirus 71 viruses by a panel of neutralizing monoclonal antibodies: current genotyping of EV71 does not reflect their antigenicity. *Vaccine*.

[37] Kiener T. K., Qiang J., Tao M., Chow V. T. K., Kwang J. (2014). A novel universal neutralizing monoclonal antibody against enterovirus 71 that targets the highly conserved “knob” region of VP3 protein. *PLoS Neglected Tropical Diseases*.

[38] Fan G., Wang Y. P., Mao Q. Y. (2012). Enterovirus 71 viral capsid protein linear epitopes: identification and characterization. *Virology Journal*.

[39] Kiener T. K., Jia Q., Lim X. (2012). Characterization and specificity of the linear epitope of the enterovirus 71 VP2 protein. *Virology Journal*.

[40] Huang M. L., Chiang P. S., Chia M. Y (2013). Cross-reactive neutralizing antibody responses to enterovirus 71 infections in young children: implications for vaccine development. *PLoS Neglected Tropical Diseases*.

[41] Tan S., Tan X., Sun X. (2013). VP2 dominated CD4+T cell responses against enterovirus 71 and cross-reactivity against coxsackievirus A16 and polioviruses in a healthy population. *The Journal of Immunology*.

[42] Arita M., Takebe Y., Wakita T., Shimizu H. (2010). A bifunctional anti-enterovirus compound that inhibits replication and the early stage of enterovirus 71 infection. *Journal of General Virology*.

[43] Mu Z., Wang B., Zhang X. (2013). Crystal structure of 2A proteinase from hand, foot and mouth disease virus. *Journal of Molecular Biology*.

[44] Castelló A., Alvarez E., Carrasco L. (2011). The multifaceted poliovirus 2A protease: regulation of gene expression by picornavirus proteases. *Journal of Biomedicine & Biotechnology*.

[45] Kuo R.-L., Kung S.-H., Hsu Y.-Y., Liu W.-T. (2002). Infection with enterovirus 71 or expression of its 2A protease induces apoptotic cell death. *Journal of General Virology*.

[46] Yang C.-H., Li H.-C., Jiang J.-G. (2010). Enterovirus type 71 2A protease functions as a transcriptional activator in yeast. *Journal of Biomedical Science*.

[47] Wen H.-L., Si L.-Y., Yuan X.-J. (2013). Complete genome sequencing and analysis of six enterovirus 71 strains with different clinical phenotypes. *Virology Journal*.

[48] Towner J. S., Ho T. V., Semler B. L. (1996). Determinants of membrane association for poliovirus protein 3AB. *Journal of Biological Chemistry*.

[49] Gao Q., Yuan S., Zhang C. (2015). Discovery of itraconazole with broad-SpectrumIn VitroAntienterovirus activity that targets nonstructural protein 3A. *Antimicrobial Agents and Chemotherapy*.

[50] Lei X., Liu X., Ma Y. (2010). The 3C protein of enterovirus 71 inhibits retinoid acid-inducible gene I-mediated interferon regulatory factor 3 activation and type I interferon responses. *Journal of Virology*.

[51] Lei X., Xiao X., Xue Q., Jin Q., He B., Wang J. (2012). Cleavage of interferon regulatory factor 7 by enterovirus 71 3C suppresses cellular responses. *Journal of Virology*.

[52] Chen S.-C., Chang L.-Y., Wang Y.-W. (2011). Sumoylation-promoted enterovirus 71 3C degradation correlates with a reduction in viral replication and cell apoptosis. *Journal of Biological Chemistry*.

[53] Kung Y. H., Huang S. W., Kuo P. H. (2009). Introduction of a strong temperature-sensitive phenotype into enterovirus 71 by altering an amino acid of virus 3D polymerase. *Virology*.

[54] Sun Y., Wang Y., Shan C. (2012). Enterovirus 71 VPg uridylation uses a two-molecular mechanism of 3D polymerase. *Journal of Virology*.

[55] Liu Y., Zheng Z., Shu B. (2016). SUMO modification stabilizes enterovirus 71 polymerase 3D to facilitate viral replication. *Journal of Virology*.

[56] Chen T.-C., Chang H.-Y., Lin P.-F. (2009). Novel antiviral agent DTriP-22 targets RNA-dependent RNA polymerase of enterovirus 71. *Antimicrobial Agents and Chemotherapy*.

[57] Kuo R.-L., Shih S.-R. (2013). Strategies to develop antivirals against enterovirus 71. *Virology Journal*.

[58] Ferrerorta C., Arias A., Escarmís C., Verdaguer N. (2006). A comparison of viral RNA-dependent RNA polymerases. *Current Opinion in Structural Biology*.

